# Functional Annotation of Hierarchical Modularity

**DOI:** 10.1371/journal.pone.0033744

**Published:** 2012-04-04

**Authors:** Kanchana Padmanabhan, Kuangyu Wang, Nagiza F. Samatova

**Affiliations:** 1 Department of Computer Science, North Carolina State University, Raleigh, North Carolina, United States of America; 2 Oak Ridge National Laboratory, Oak Ridge, Tennessee, United States of America; 3 Bioinformatics Research Center, North Carolina State University, Raleigh, North Carolina, United States of America; Beijing Normal University, Beijing, China

## Abstract

In biological networks of molecular interactions in a cell, network motifs that are biologically relevant are also functionally coherent, or form functional modules. These functionally coherent modules combine in a hierarchical manner into larger, less cohesive subsystems, thus revealing one of the essential design principles of system-level cellular organization and function–hierarchical modularity. Arguably, hierarchical modularity has not been explicitly taken into consideration by most, if not all, functional annotation systems. As a result, the existing methods would often fail to assign a statistically significant functional coherence score to biologically relevant molecular machines. We developed a methodology for hierarchical functional annotation. Given the hierarchical taxonomy of functional concepts (e.g., Gene Ontology) and the association of individual genes or proteins with these concepts (e.g., GO terms), our method will assign a Hierarchical Modularity Score (HMS) to each node in the hierarchy of functional modules; the HMS score and its 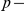
value measure functional coherence of each module in the hierarchy. While existing methods annotate each module with a set of “enriched” functional terms in a bag of genes, our complementary method provides the hierarchical functional annotation of the modules and their hierarchically organized components. A hierarchical organization of functional modules often comes as a bi-product of cluster analysis of gene expression data or protein interaction data. Otherwise, our method will automatically build such a hierarchy by directly incorporating the functional taxonomy information into the hierarchy search process and by allowing multi-functional genes to be part of more than one component in the hierarchy. In addition, its underlying HMS scoring metric ensures that functional specificity of the terms across different levels of the hierarchical taxonomy is properly treated. We have evaluated our method using *Saccharomyces cerevisiae* data from KEGG and MIPS databases and several other computationally derived and curated datasets. The code and additional supplemental files can be obtained from http://code.google.com/p/functional-annotation-of-hierarchical-modularity/ (Accessed 2012 March 13).

## Introduction

Network motifs are recurring, statistically significant patterns of node interactions that act as building blocks of complex networks [1]. In biological networks of molecular interactions in a cell, such as protein-protein interaction (PPI) networks or gene transcriptional regulatory networks (TRN), network motifs that are biologically relevant are also *functionally coherent*, or form *functional modules* [2], such as a ribosomal module synthesizing proteins or a signal transduction system governing bacterial chemotaxis. These functionally homogenous modules combine in a hierarchical manner into larger, less cohesive subsystems, thus revealing one of the essential design principles of system-level cellular organization and function–*hierachical modularity* [3], [4].

Hierarchical modularity manifests itself at various levels of cellular organization. At the metabolism level, for example, hierarchical modularity within *Escherichia coli* closely overlaps with known metabolic functions, such as pyrimidine metabolism [3].

At the regulation level, for instance, in the *E. coli* TRN network, network motifs without global regulators, such as feed forward loops and bi-fan motifs, form the multi-layered hierarchical structure without feedback regulation [5]. Analysis of the hydrogen-producing *Rhodopseudomonas palustris* transcriptome [6] also suggests the interplay between functionally coherent modules related to electron transport (*fixX, fixC, fixB, fixA, ferN, fer1*), co-factor synthesis (*nifB, nifV, nifQ, nifN, nifE, nifX*), assembly or stability (*nifW, nifS2, nifU*), and regulation (*nifA*). Likewise, the CD4+ T-cell modules involved in human immune protection and regulation are made up of polarizing cues, lineage-specifying transcription factors, homing receptors, and effector molecules [7].

At the protein-protein interaction level, the discovered functional modules in the *Saccharomyces cerevisiae* PPI network consist of sub-components in the form of protein complexes and other macro-molecular assemblies [8]. For instance, the DNA replication, chromosome segregation, and chromatin assembly module consists of several submodules including DNA repair, DNA replication, chromosome segregation, origin recognition complex, anaphase promoting complex, spindle pole body, and chromatin assembly [9].

Thus, these examples provide a strong support not only for the network modularity principle introduced by Hartwell *et al.* [2] but also for the hierarchical modularity as a generic principle of system-level cellular organization and function [3].

Arguably, hierarchical modularity has not been explicitly taken into consideration by most, if not all, functional annotation systems [10], [11]. Instead, a functional module is traditionally viewed as a “*bag of genes*,” and methods that assess its functional coherence, or provide functional annotation, analyze this bag in its entirety. As a result, the existing methods would often fail to assign a statistically significant functional coherence score to biologically relevant molecular machines (see Table 1).

**Table 1:** Statistical significance of protein pairs' functional coherence in *Saccharomyces cerevisiae*.

Protein pair				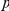 -value (pair/module/module size)					Ref.
ID	Description	ID	Description	HMS	[20], [21]	[25]	[26]	[27], [28]	
*SNU13*	RNA binding protein	*DIB1*	17-kDa component of the U4/U6aU5 tri-snRNP	0.0/0.0/2	0.23	0.01/0.01/2	0.1/0.1/2	0.42/0.42/2	[56]
*HAP1*	Zinc finger transcription factor involved in the complex regulation of gene expression in response to levels of heme and oxygen	*RPM2*	Protein subunit of mitochondrial RNase P	0.0/0.01/3	0.214	0.1/0.337/3	0.02/1.0/3	0.51/1.0*/3	[57]
*SRB2*	Subunit of the RNA polymerase II mediator complex	*RPB9*	RNA polymerase II subunit B12.6	0.0/0.01/58	0.48	0.12/1.0*/58	0.333/1.0/58	0.98/1.0*/58	[55]
*NSR1*	Nucleolar protein that binds nuclear localization sequences	*DBP2*	Essential ATP-dependent RNA helicase of the DEAD-box protein family	0.0/0.0/2	0.44	0.1/0.1/2	0.13/0.13/2	0.74/0.74/2	[58]

* assigned a 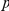-value of 1 because the tool was unable to find a score for the entire module.

To address this gap, we developed a methodology for hierarchical functional annotation of biological network motifs. Given the hierarchical taxonomy of functional concepts (e.g., Gene Ontology) and the association of individual genes or proteins with these concepts (e.g., GO terms), our method will assign a *Hierarchical Modularity Score* (**HMS**) to each node in the hierarchy of functional modules; the HMS score and its 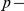
value measure functional coherence of each module in the hierarchy. While existing methods annotate each module with a set of “enriched” functional terms in a bag of genes, our complementary method provides the hierarchical functional annotation of the modules and their components that are hierarchically organized.

A hierarchical organization of functional modules often comes as a bi-product of cluster analysis of gene expression data or protein interaction data. Otherwise, our method will automatically build such a hierarchy by directly incorporating the functional taxonomy information into the hierarchy search process and by allowing multi-functional genes to be part of more than one component in the hierarchy. In addition, its underlying HMS scoring metric ensures that functional specificity of the terms across different levels of the hierarchical taxonomy is properly treated.

We have evaluated our method using *Saccharomyces cerevisiae* data from KEGG [12]–[14] and MIPS [15] and several other computationally derived and curated datasets [8], [16]–[19]. We compared our method with several biological significance analysis methods [20]–[28]. The hierarchical modularity built by our method from a set of genes in various KEGG pathways produces biologically relevant modules, namely, at various levels of the hierarchy, the corresponding modules match quite well with the manually-curated hierarchy of pathways in KEGG. We have obtained similar results for the protein complexes in the MIPS database. We provide literature evidence for several functional modules that have been identified by HMS as signicant both at the protein pairs and at the module levels but have been missed by some existing methods.

## Results

### Benchmark data and tools

To evaluate the performance of our method, we first need to define (1) the model organism; (2) the benchmark data of known functional annotations for this organism; (3) the hierarchical taxonomy of functional terms, and (4) the state-of-the-art methods that are most suitable for our comparative analysis.

*Saccharomyces cerevisiae* is our model organism. The reason is that its genome annotation is mostly complete and manually curated by human experts [22]. Apart from annotation quality, the availability of functional module datasets, both manually curated and experimentally generated, for *S. cerevisiae* is advantageous for our method validation purposes.

For benchmark data, we plan to use both metabolic pathways from KEGG database [12]–[14] and protein complexes in MIPS database [15] including experimental protein-protein interaction data and protein complexes derived from this data [8], [16]–[19].

For the hierarchical taxonomy of functional terms, we will rely on the commonly-used functional annotation taxonomy provided by the Gene Ontology consortium [10]. As such, we will limit ourselves to the existing methods that are also based on GO ontology. Namely, we will compare our method with the ones by Pandey *et al.* [20], [21], Chen *et al.* [22], and GS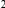 [23] methods. The former makes use of the lowest common ancestor principle to score functional coherence for a protein pair; it is based on Jiang and Conrath's scoring method [29], which is a normalized version of the scoring method in Resnik *et al.* [30]. It has been shown that Jiang and Conrath's method is the best measure to capture *semantic relatedness* [31]. The method by Chen *et al.* is based on a widely used *cosine* similarity measure to assess functional coherence for a protein pair and the authors provide a Matlab implementation for the same. The 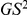 [23] uses the overlap similarity measure, and the authors provide a Python implementation for the same. Additionally, we perform comparisons with methods described in [23]–[28]. These methods [24]–[28] have web-based implementations. The 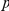-value for our method is calculated using the Monte Carlo procedure [32] and is discussed in detail in the *Methods* section.

We conducted three major types of performance evaluation: (1) at the level of functional coherency for protein pairs; (2) at the level of functional coherency for protein functional modules (with two or more proteins in each); and (3) functional annotation of reconstructed hierarchical functional modules. Both large-scale comparative analysis and small-scale literature mining based validation are performed.

### Functional coherency of protein functional modules

#### Detailed biological analysis of modules from Chen and Yuan [8]

**Figure 1:**
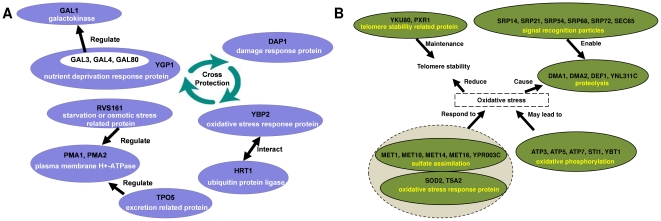
Functionally coherent modules from the Chen and Yuan [**8**] study. (A) Module ID M1 and (B) Module ID M3.

Two functional modules, M1 (Figure 1.A) and M3 (Figure 1.B), with the same ID's as in [8], have been reported as insignificant by several existing functional enrichment analysis methods (see Table 2). We used the web-based implementations for the functional enrichment analysis methods. However, the modules were identified as significant by our HMS method. In the following paragraphs, we provide biological evidence for the subtrees in the functional hierarchy of the two modules.

**Table 2:** Functional modules evaluated using existing enrichment analysis tools in comparison with HMS.

	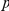 -value			
Module ID	[25]	[27], [28]	[26]	HMS
M12	1.02E-12	3.4E-19	5.73E-01	0.00
M94	1.05E-07	4.0E-7	6.03E-04	0.00
M3	1.0	0.1	1.0	0.02
M1	1.0	0.18	1.0	0.04

The first two rows show two homogeneous functional modules and the next two rows of the table show heterogeneous functional modules that have coherent submodules. Functional modules have been obtained from Chen and Yuan [8] of the *Saccharomyces cerevisiae* PPI network.

In module M1 (see Figure 1.A), *GAL1* is the galactose structural gene and *GAL3*, *GAL4*, and *GAL80* are transcriptional regulators involved in activation of the *GAL* genes in response to galactose; they form a sub-module in the hierarchy. The pair-wise functional associations between these genes are well-documented. Transcription of the galactose pathway genes in *Saccharomyces cerevisiae* (*S. cerevisiae*) and *Kluyveromyces lactis* (*K. lactis*) is induced by galactose through the activities of the regulatory proteins, *GAL4*, *GAL80*, and *GAL3* (*S. cerevisiae*) or *GAL1* (*K. lactis*) [33], [34]. *GAL4* binds to its binding sites in both the absence and the presence of galactose [35]; it has the capacity to activate transcription, while *GAL80* inhibits *GAL4* in the absence of galactose [36]. At the presence of galactose, *GAL3* (*GAL1* in *K. lactis*) binds to *GAL80* that alleviates the inhibition effect of *GAL80* upon *GAL4* [37].

*PMA1* and *PMA2* form another sub-module that encodes plasma membrane H+-ATPase (PM-H+-ATPase), an enzyme with critical physiological roles both in the absence or presence of environmental stress. *PMA2*, showing 89% identity to *PMA1* at the amino acid sequence level, encodes an H+-ATPase that is functionally interchangeable with the one encoded by *PMA1* [38].

The third sub-module involves *DAP1*, the damage response protein, and *YGP1* induced by nutrient deprivation-associated growth arrest. *DAP1* is required for growth in the presence of the methylating agent methyl methanesulfonate (MMS). *DAP1* is required for cell cycle progression following damage [39], while *YGP1* is induced after exposing cells to nutrient limitation [39]. It has already been demonstrated that exposure to one kind of stress can activate protective mechanisms against other different stresses, a phenomenon known as cross-protection [40]. Since *DAP1* and *YGP1* act both in the process of stress response, cross-protection might associate these two genes together.

The same relationship based on cross-protection can be observed in another sub-module that consists of *YBP2* that plays the role in resistance to oxidative stress and *HRT1* that is involved in stress response. The transcription factor *YBP2* and its homologue play central roles in the determination of resistance to oxidative stress [41], while *HRT1* forms ubiquitin ligase complex with other scaffold proteins [42]. The critical stress response factor *Nrf2* has been shown to be repressed by the ubiquitin-proteasome system under normal, unstressed conditions, with *Nrf2* exploiting ubiquitin ligase complexes [43].

The next module is made up of *PMA1* and *TPO5* that are involved in excretion of putrescine and spermidine. *TPO5* functions as a suppressor of cell growth by excreting polyamines [44]. *PMA1* is a polytopic membrane protein, whose essential physiological function is to pump protons out of the cell. Both the excretion of putrescine by *TPO5* and the delivery of *PMA1* to cell surface rely on secretory pathway. Furthermore, small portions of *TPO5* are co-localized with *PMA1* in plasma membrane, which indicates possible interactions between these two proteins [45].

*PMA1* also forms a sub-module together with *RVS161* that regulates polarization of the actin cytoskeleton. *RVS161* regulates secretory vesicle trafficking [46] as well as cell polarity [47], actin cytoskeleton polarization [48], and endocytosis [49]. It is already known that the efficient delivery of *PMA1* to cell surface relies on secretory pathway [45]. Thus, *RVS161* has a regulatory effect upon *PMA1*.

Genes in this module have coherent functions, namely more than half of the proteins in this module are related to stress response, five out of 20 total have regulatory roles in cell cycle, four out of 20 total are evolved in endocytosis. Stress conditions are likely to cause cell cycle arrest, as well as endocytosis induction.

For module M3 (see Figure 1.B), the vast majority of the genes in the module enjoy oxidative stress response as the common theme. *SOD2* protects cells against oxygen toxicity and *TSA2*, responsible for the removal of reactive oxygen, directly protects cells against oxidative stress, while *PXR1* plays the role in negative regulation of telomerase, and *YKU80*, a subunit of the telomeric Ku complex, contributes to the maintenance of telomere stability, since oxidative stress is likely to induce telomere attrition [50].

Meanwhile, proteolysis could also be the result of oxidative stress: *YNL311C* is part of an ubiquitin protease complex, *DEF1* enables ubiquitination, *DMA1* is involved in ubiquitin ligation, and *DMA2* is involved in ubiquitination [51]. Since proteolysis involves many protein transportation processes, the signal recognition particles are essential to enable transportation: *SRP14*, *SRP21*, *SRP54*, *SRP68*, *SRP72*, and *SEC65* are all part of the signal recognition particle (SRP) subunit, and appear in module M3.

Furthermore, Wu *et al.* [52] showed that repression of sulfate assimilation is an adaptive response of yeast to the oxidative stress of zinc deficiency, while we notice that *MET1*, *MET10*, *MET14*, *MET16*, and *YPR003C* are basic proteins or protein subunits that are required for sulfate assimilation. Finally, oxidative phosphorylation produces ATP by utilizing electron transport trains. As a result, the inhibition of electron transport chain will lead to oxidative stress [53]. That is probably why *ATP3*, *ATP5*, and *ATP7* are all part of the enzyme complex required for ATP synthesis. Also, *STI1*, ATPase inhibitor activity, and *YBT1*, ATPase activity, coupled to transmembrane movement of substances, are part of the module.

#### Large-scale analysis of protein functional modules

Protein functional modules predicted by Chen *et al.* using their betweenness-based network partitioning algorithm [8] and protein complexes from CYS2008 database [19] are analyzed as modules for their functional coherency. Table 3 summarizes the results obtained by our HMS scoring method and GS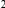 [23] method for both *significant* (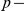
value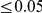) and *highly significant* (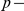
value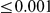) cut-offs. HMS predicted 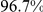 of the CYS complexes and 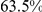 of the modules from Chen and Yuan study to be significant. GS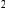 predicted 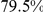 of the CYS complexes and 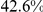 of the modules from Chen and Yuan [8] to be significant. The results can be found in Supplement S1.

**Table 3:** Percentage of significant (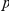-value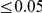) and highly significant (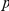-value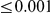) functionally coherent modules from Chen and Yuan [8] and CYS2008 [19].

Dataset	Method	Significant	Highly significant
CYS2008 protein complex database [19]	HMS	96.7%	82.9%
	GS 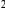 [23]	79.5%	40.1%
Chen and Yuan [8]	HMS	63.5%	46.4%
	GS 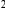 [23]	42.6%	29.8%

#### HMS comparison with protein-set semantic similarity scoring metric

**Figure 2:**
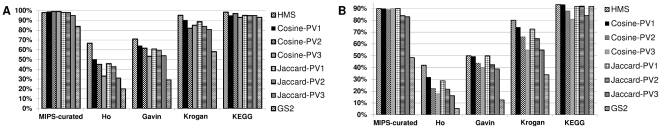
Functional coherence analysis of protein complexes and pathways. Functional coherence analysis of protein complexes from MIPS-curated [15], Ho [17], Gavin [18], and Krogan [16] as well as metabolic pathways from KEGG. Comparison between our HMS scoring, cosine similarity with different 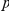-value methods from [22], Jaccard similarity with different 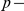
value methods from [22] and GS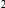 [23] methods. (A) Significant Modules (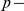
value
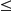
0.05 and (B) Highly Significant Modules (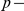
value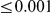).

Protein pairs from the same protein complexes in [15]–[18] or the same metabolic pathways in KEGG [12]–[14] are assessed for functional coherency using HMS scoring method, the cosine similarity metric [22], the Jaccard similarity metric [22], and the GS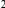 [23] method. We filter our results as being *significant* (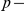
value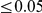, Figure 2.A) and *highly significant* (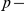
value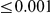, Figure 2.B).

For MIPS-curated data [15], HMS, cosine, and Jaccard methods predicted nearly 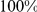 of the protein functional modules as being functionally coherent, while GS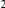 only predicted 84% to be significant. For Ho *et al.* data [17], HMS, on average, provided 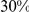 higher predictions than other methods. For Krogan *et al.* data [16], HMS performed 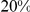 better than the other methods, on average. For KEGG data, our HMS method, on average, performed 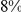 better than the other methods. For Gavin *et al.* data [18], HMS performed about 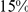 better, on average. Additionally, Chagoyen *et al.* [22] mentioned some complexes and pathways that in spite of being functionally related were predicted incoherent. We list some of those modules in Table 4 and show that our HMS method is able to predict them as functionally related. The results can be found in Supplement S1.

**Table 4:** HMS results for some KEGG metabolic pathways and MIPS protein complexes [22] classified as insignificant by Chagoyen *et al.* [22].

		Chagoyen *et al.* [22]			HMS	
Pathway or Complex Name	Size	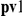	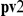	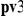	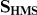	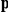 -value
DNA helicases	2	4.12E-01	5.36E-01	5.36E-01	0.21	0.0
Mitochondrial processing complexes	4	1.05E-01	1.46E-01	2.73E-01	0.35	0.0
Tryptophan metabolism	16	7.67E-02	4.12E-01	5.08E-01	0.34	0.0
Lipoic acid metabolism	3	4.09E-01	4.69E-01	4.69E-01	0.50	0.0
Limonene and pinene degradation	6	2.59E-01	4.13E-02	1.66E-01	0.30	0.0

### Functional coherency of protein pairs

#### HMS comparison with pair-wise semantic similarity metrics

We also calculated HMS score for 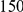 functionally-associated protein pairs and compared these scores with the HMS scores for an equal number of non-functional protein pairs in *S. cerevisiae*. The former were obtained from STRING [54] with a strong functional association score of 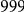 out of 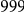. The latter were sampled from those pairs that were not scored in STRING (i.e., there is no evidence for their functional association). We also performed a similar analysis using four other pair-wise protein similarity scores, Pandey *et al.* [20], [21] metric, GS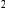 [23] metric, overlap score [24], and cosine similarity [22], [24]. The results of the analysis are summarized in Table 5. For all methods, the mean score for the functionally-associated pairs is significantly different from the mean score for the non-functional pairs, but HMS has the lowest 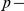
value. Additionally, we calculated the percentage of the total number of pairs whose score is lower than the maximum score of the non-functional pairs but greater than the minimum score of the functionally-associated pairs. We found that except for HMS and Pandey *et al.* [20], [21], all the other methods have an overlap. This is one of the reasons why we selected Pandey *et al.* [20], [21] method for comparison in the next section. The results can be found in Supplement S1.

**Table 5:** Comparison of pair-wise semantic similarity metrics using functionally-associated and non-functional protein pairs.

Method	Ref.	Functionally-associated Pairs			Non-functional Pairs				
		Mean	Std	Median	Mean	Std	Median	Overlap (%)	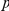 -value
HMS		0.56	0.06	0.53	0.02	0.03	0.0	0	3.89E-61
GS 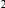	[23]	0.78	0.18	0.79	0.38	0.13	0.36	63.2	8.42E-74
Pandey	[20], [21]	0.71	0.23	0.73	0.07	0.02	0.06	0	6.53E-29
Overlap Score	[24]	0.91	0.13	1.00	0.41	0.33	0.25	54.8	8.59E-40
Cosine similarity	[22], [24]	0.78	0.17	0.80	0.20	0.08	0.20	6	3.05E-94

We analyzed some functionally-associated protein pairs from STRING that were classified as functionally coherent and thus biologically relevant (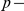
value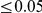) by our method, yet were assessed as incoherent by Pandey's *et al.* We found literature support for biological relevance of these protein pairs. The results are summarized in Table 1. *RPB9* and *SRB2* proteins are part of *RNA polymerase II holoenzyme* in *S. cerevisiae* [55]. *SNU13* and *DIB1* proteins have been shown to be associated with the U4/U6U5 pre-mRNA splicing small nuclear ribonucleoprotein (snRNP) complex [56]. *HAP1* and *RPM2* are related by the fact that *RPM2* is required for repression of the heme activator protein *HAP2* in the absense of heme [57]. When *NSR1* was used as a bait in the protein-fragment complementation assay (PCA), the experiment pulled out *DBP2* as one of its prey proteins [58].

### Inferred hierarchy of functional modules

To assess the quality of the hierarchy of functional modules derived from a given “bag of genes” using our HMS scoring metric and the hierarchical modularity inference methodology described in the *Methods* section, we assess the consistency between the predicted hierarchy and the hierarchy of known functional concepts in KEGG and MIPS databases. Remind that HMS, by default, uses GO ontology as its hierarchical taxonomy of functional terms.

#### Consistency analysis for KEGG metabolic pathways

Note that each metabolic pathway is a functional module. We consider the genes from several metabolic pathways as one “bag of genes” to build the hierarchy of functional modules. If the constructed hierarchy of functional modularity is biologically relevant, then the genes in each pathway should form a subtree in the hierarchy and not be “contaminated” by the genes from the other pathways. We set the fuzziness to null before running the algorithm in order to be able to use standard clustering validation metrics like the Heidke Score [59], Gerrity Score [60], and Peirce Score [61].

Since KEGG is organized into a three level hierarchy, the pathways at the lower levels of the hierarchy are functionally more coherent. Hence, they should be harder to separate into different subtrees. This hierarchical specificity of the KEGG knowledgebase provides us with an opportunity to check both the specificity and the sensitivity of our hierarchical modularity inference method.

We build contingency tables to provide a mathematically and statistically sound way for assessing the performance at large-scale. To construct a contingency table, the inferred hierarchy is first cut at the level that produces 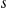 subtrees that are then compared with 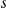 pathways used as input to the algorithm. In the ideal scenario, all the genes in a given pathway (or row in the contingency table) will end up in the corresponding subtree (or the column in the contingency table) and vice versa; or the contingency table will form a diagonal matrix with the number of pathway genes along the diagonal and zero's on the off-diagonal elements of the table. By completing such a contingency table, we could then utilize various skill metrics, such as Heidke Score [59], Gerrity Score [60], and Peirce Score [61], to measure the goodness of the predicted hierarchical modularity.

We also performed all the experiments by replacing the 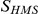 scoring metric with the one proposed by Pandey *et al.* [20], [21] and compiled the results in Table 6. We found that at “Level 1” in the KEGG hierarchy, both methods had a perfect score of 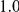 for all three metrics, but as we moved down the hierarchy, we found that our method performed consistantly better than Pandey's *et al.* [20], [21]. At “Level 2,” we found that our method performed 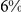, 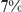, and 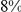 better in terms of the Heidke score, the Pierce score, the Gerrity score, respectively. At “Level 3,” which is probably the hardest of the three in terms of pathways seperability, we performed about 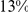, 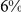, and 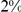 better for the same skill metrics. The results can be found in Supplement S2.

**Table 6:** Skill metrics for *Saccharomyces cerevisiae* KEGG experiments.

	KEGG	Heidke Score	Pierce Score	Gerrity Score
HMS	Level 1	1 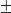 0	1 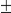 0	1 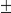 0
[20], [21]		1 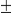 0	1 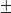 0	1 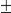 0
HMS	Level 2	0.97 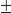 0.04	0.98 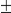 0.05	0.98 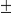 0.05
[20], [21]		0.91 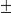 0.1	0.91 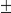 0.12	0.90 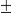 0.12
HMS	Level 3	0.90 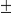 0.03	0.90 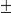 0.05	0.90 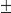 0.06
[20], [21]		0.77 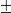 0.14	0.84 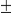 0.10	0.88 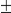 0.07

#### Consistency analysis for MIPS protein complexes

Protein complexes are functionally coherent modules, and hence experiments similar to the ones preformed using KEGG pathways can be designed. The results can be found in Table 7. We compared the mean score reported for our method and the one proposed by Pandey *et al.* We found that at “Level 1” in the MIPS hierarchy, our method performed 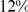 better than Pandey's *et al.* for both the Heidke and Pierce scores and 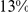 better for the Gerrity score. At “Level 2,” our method performed approximately 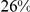 better in terms of the Pierce Score and 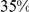 and 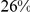 better in terms of the Heidke and Gerrity scores, respectively. The results can be found in Supplement S2.

**Table 7:** Skill metrics for *Saccharomyces cerevisiae* MIPS experiments.

	MPact-MIPS	Heidke Score	Pierce Score	Gerrity Score
HMS	Level 1	1 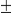 0	1 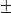 0	1 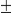 0
[20], [21]		0.88 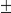 0.25	0.88 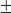 0.25	0.87 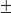 0.26
HMS	Level 2	0.89 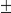 0.14	0.90 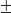 0.13	0.90 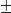 0.13
[20], [21]		0.54 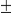 0.37	0.64 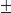 0.27	0.64 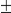 0.27

### Effect of fuzziness

To evaluate the effect of incorporating fuzziness into the reconstruction of hierarchical modularity, we selected several KEGG pathways with common genes and then reconstructed the hierarchy with the fuzziness parameter 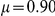. For each pathway, we identified the corresponding cluster with the maximum gene overlap (at least 75%). We analyzed multi-pathway genes in terms of their membership in the corresponding clusters. Table 8 summarizes the results of the analysis for multi-pathway genes. Except for *UGA1* gene, which was missed in the cluster corresponding to the *Valine, leucine and isoleucine degradation* pathway, all the other genes were properly identified in their corresponding clusters.

**Table 8:** Consistency of multi-pathway genes across clusters that enrich the corressponding pathways.

	Genes								
Pathways	*ALD4*	*ALD5*	*ALD6*	*ERG10*	*ERG13*	*SHM1*	*SHM2*	*UGA1*	*POX1*
Propanoate metabolism	1/1	1/1	1/1	1/1	0/0	0/0	0/0	1/1	0/0
Valine, leucine and isoleucine degradation	1/1	1/1	1/1	1/1	1/1	0/0	0/0	1/0	0/0
Cyanoamino acid metabolism	0/0	0/0	0/0	0/0	0/0	1/1	1/1	0/0	0/0
Methane metabolism	0/0	0/0	0/0	0/0	0/0	1/1	1/1	0/0	0/0
beta-Alanine degradation	1/1	1/1	1/1	0/0	0/0	0/0	0/0	1/1	0/0
Synthesis and degradation of ketone bodies	0/0	0/0	0/0	1/1	1/1	0/0	0/0	0/0	0/0
Lysine degradation	1/1	1/1	1/1	1/1	0/0	0/0	0/0	0/0	0/0
Biosynthesis of unsaturated fatty acids	0/0	0/0	0/0	0/0	0/0	0/0	0/0	0/0	1/1
Fatty acid metabolism	1/1	1/1	1/1	0/0	0/0	0/0	0/0	0/0	1/1

### Choosing 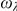 value

**Figure 3:**
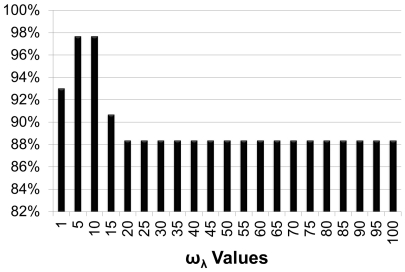
Effect of different values of 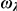 on the 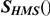 score.

Our 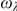 selection strategy aims to optimize the method performance on a validation set of protein complexes, that are essentially known functional modules (Figure 3). This prior knowledge is derived from manually curated set of complexes from MPact-MIPS [15] database. Starting with the most conservative value of 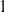 for the 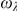 value, for each 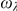 value, we calculate the accuracy of identifying known protein complexes from the validation set as being statistically significant. We pick a value that is lenient enough to classify most of the known functional modules (manually curated protein complexes) as significant, while being stringent enough to avoid predicting random modules from getting high 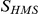 scores. Thus, we select the largest 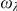 (in this case (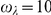)) value that ensures that at least 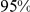 of the validation protein complex set is predicted as being statistically significant. The significance of a protein complex score is calculated using the Monte Carlo method discussed in the *Methods* section (using a 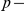
value threshold of 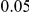).

## Discussion

*Functional coherency analysis* and *functional enrichment analysis* are two important concepts in genome annotation. Functional coherency analysis assesses if a set of genes or proteins are biologically relevant. Functional enrichment analysis determines if the distribution of a functional term in the set of genes is significantly different from the distribution of the same functional term in a background set of genes. Thus, functional coherency analysis scores a *functional module*, whereas functional enrichment analysis scores a *functional term*.

With functional enrichment analysis, it is sometimes difficult to conclude whether the set of genes is coherent. For example, for a set of 14 genes, one Gene Ontology (GO)-based functional enrichment analysis tool could infer that 9 out 14 genes are enriched with *GO term A* with a 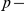
value of 0.001 and 11 out of 14 genes are enriched with *GO term B* with 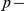
value of 0.434. Such inference creates ambiguity in deciding whether the set of genes is coherent. Therefore, it is functional coherence analysis that often determines whether the given functional module is biologically significant.

Most existing functional enrichment analysis methods [25], [26], [62]–[70] assume that proteins in the same functional module perform the same function, or they are *functionally coherent*. Hence, for functionally coherent modules, all the proteins are annotated with the same functional term.

One of the main limitations of the existing functional enrichment analysis methods is that they require the use of a *background set of annotated genes*. As discussed by Shah and Fedoroff [63], the background could severely affect the assigned 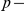
value, because this background information is directly incorporated into the scoring mechanism. Thus, functional enrichment scores that require a background set must be interpreted with caution. Unlike these functional enrichment analysis methods, we analyze functional coherency of proteins in the functional module without incorporating a *prior* knowledge about a background set into the scoring metric.

Both functional coherency analysis and functional enrichment analysis often rely on functional annotation taxonomies, such as the Gene Ontology (GO) [10] or FunCat [11] that are *hierarchical* by nature. Hence, any protein or gene associated with the child node is also associated with the parent node in the taxonomy. As discussed by Khatri and Dr
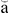
ghic [64], some tools [65]–[67] only utilize direct annotations, or functional terms associated with the child node. Yet, other tools [71]–[73] use functional terms associated only with the nodes at the user-specified hierarchical level; the more specific functional terms associated with the nodes below this level are replaced with a more generic parent's term at the user-defined level. Likewise, some methods take into consideration only the parent's term but not all its ancestors' [74]. And other tools use both [25], [26], [62], [67], [75]–[77]. Unlike these methods, we take all the levels of the hierarchy (a node and all its ancestors) into consideration while assessing a module for its functional coherence.

One of the drawbacks of these hierarchical taxonomy-based tools is their inability to differentiate between the functional terms that directly annotate the gene and those that annotate its ancestors; basically, they assign the same weight to both. Some methods [68], [69] make this differentiation but in a statistically non-sound manner [69]. Unlike these methods, we utilize the hierarchical taxonomy of functional terms in its entirety, by discriminating between direct annotations and those associated with gene's ancestors.

Existing functional coherency analysis methods including the ones by Pandey *et al.* [20], [21] and Chagoyen *et al.* [22] assess functional coherence of a pair or of a set of genes using *GO term* annotations. The methods by Pandey *et al.* [20], [21] are heavily based on Resnik's [30] information-theoretic score and its extension by Lin *et al.* [78]. This scoring incorporates the functional term's distribution for the background set directly into the scoring. The method by Chagoyen *et al.* [22] utilizes the cosine similarity to assess functional similarity between a pair of proteins. The number of functional terms that the protein is annotated with affects the scoring; multi-functional proteins will probably have more 1's in their term-vector. The method weighs the term's specificity but the weighting scheme is still based on the distribution of the term in the background set, and thus it has its drawbacks. In addition, these methods do not annotate the functional module. Unlike these methods, we do not measure functional specificity based on any background set. We calculate the specificity based on the position of a node in the hierarchical functional taxonomy. We also provide functional annotation for the module.

An important requirement for a good functional coherence scoring method is its ability to distinguish the number of levels traversed in the taxonomy to identify the common ancestor for a pair of proteins. This requirement is currently not incorporated into any of the existing methods. Also, the existing techniques assess functional coherency of the module in its entirety and do not take into consideration any structural information of the module. In contrast, our method addresses those and some other limitations.

The methods discussed so far directly rely on functional annotation taxonomies. In contrast, there are methods [79]–[81] that suggest mining biomedical literature and infer functional similarity of proteins in the set based on the semantic similarity of biological concepts, or topics, covered by various literature sources that reference these proteins. In this regard, these methods are complementary to the aforementioned ones. They are particularly suitable for gene sets with missing annotations (e.g., no GO terms are assigned). They could also be used for validating and/or comparing against the GO-based inference methodologies. It is worthwhile observing that, while biomedical literature is abundant, the analysis quality is dependent on the quality of the literature used. Additio'nally, some organisms are more heavily studied than others, and hence protein sets may be evaluated as insignificant purely on the basis that the knowledge about that set is not yet available. This problem can be compared to the problem of incomplete annotations of certain genomes, and hence the disadvantage of using functional annotation taxonomies is also present here.

The number of functional modules output by a computational method is in the order of hundreds and all of them cannot be tested via experimenatation by a biologist. Hence, functional coherence and significance methods can help narrow down the search space by only selecting the most promising modules. Our method goes one step further in that, given a hierarchical module it provides a global functional view, i.e., the entire picture about all the functions within a module and suggests clues on how various submodules within the module could relate to each other. Additionally, it scores the module keeping in mind the existing hierarchical structure.

A well-known hierarchical modularity principle suggests that protein modules are hierarchically organized; multi–functional proteins further suggest that such modules could be overlapping. Moreover, hierarchical taxonomies of functional terms manifested by GO ontology or by KEGG knowledgebase further suggest a possible hierarchical functional organization of the consituent submodules of the target module. Hence, given a *bag of genes* as a functional module, our method recreates its putative hierarchical functional view, while taking into consideration the fact that some proteins could be multi–functional. This kind of functional hierarchy could help with understanding the functioning of the module at various levels of functional specificity. For example, the overall function of the target module could be *chromosome segregation*, but at lower level of the functional hierarchy, we could find a submodule responsible for *proper alignment and attachment of chromosomes* and another submodule responsible for *translating the force generated by microtubule depolarization into movement to facilitate chromosome segregation* [8].

Additionally, our functional coherence method scores each submodule and uses this information to score the overall functional coherency of the module. Building the functional hierarchy for *a bag of genes* in a target module could additionally provide a clearer picture about the core and peripheral proteins for the functioning of this module. Since the method allows *fuzziness*, the core protein's interaction with a peripheral protein (that may not interact with any other protein in the module) could thus be captured. For example, if the “bag of genes” contains *CHD1, RAD16, VPS1, NHP10, ISW2, NHP6B, ISW1*, and *RSC6*, then the core of this module could include *CHD1, VPS1, NHP10, ISW2, NHP6B*, and *ISW1* [82], while the peripheral protein *RAD16* [82] could be functionally associated only with *VPS1* and the peripheral protein *RSC6* [82] could be associated with *ISW1*. Such information could thus be explicitly captured in the hierarchy, because both *VPS1* and *ISW1* could be associated with the core and the peripheral proteins when “fuzziness” is allowed.

## Methods

**Figure 4:**
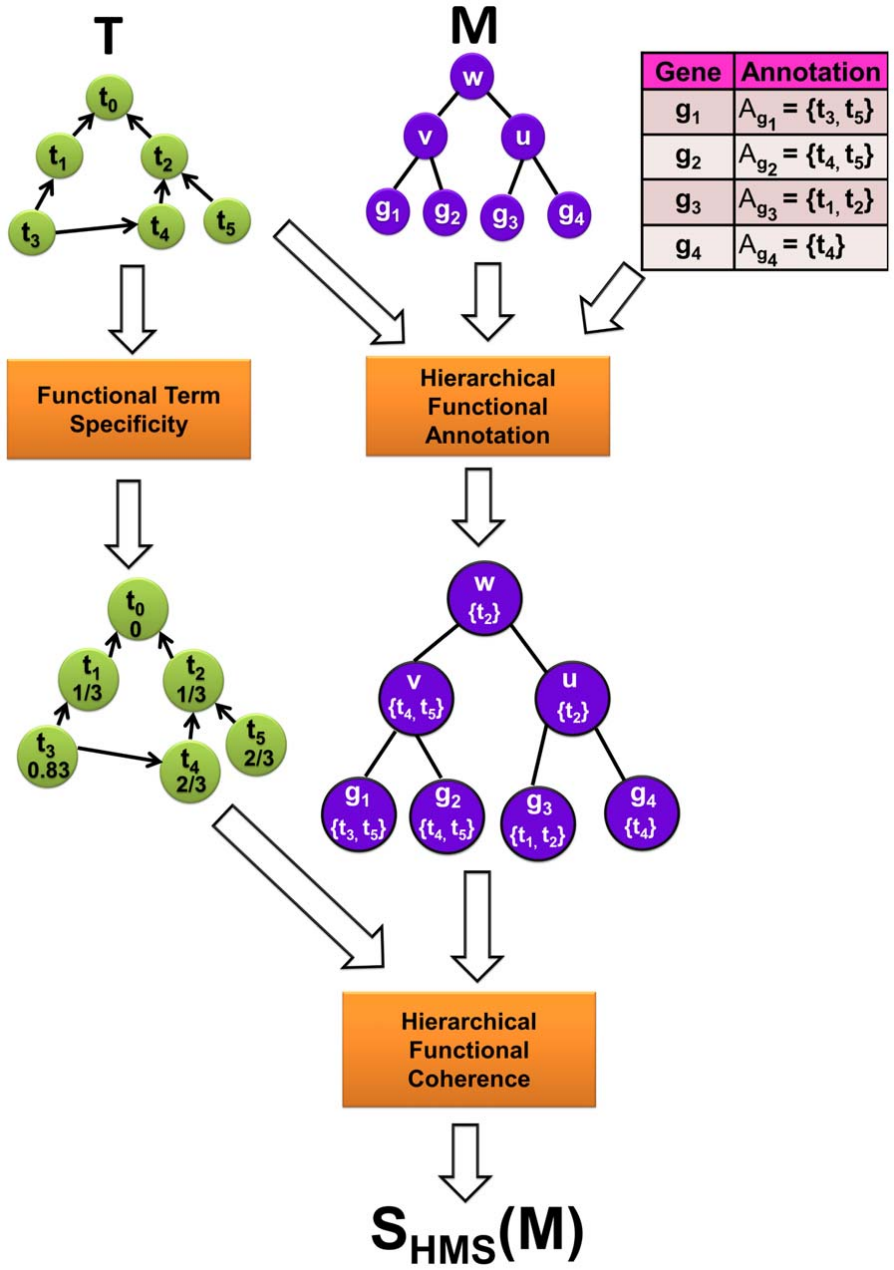
Functional annotation and coherence of hierarchical modules. The figure shows the overview of the methodology to assess functional coherence and assign annotation to hierarchical functional modules.

**Figure 5:**
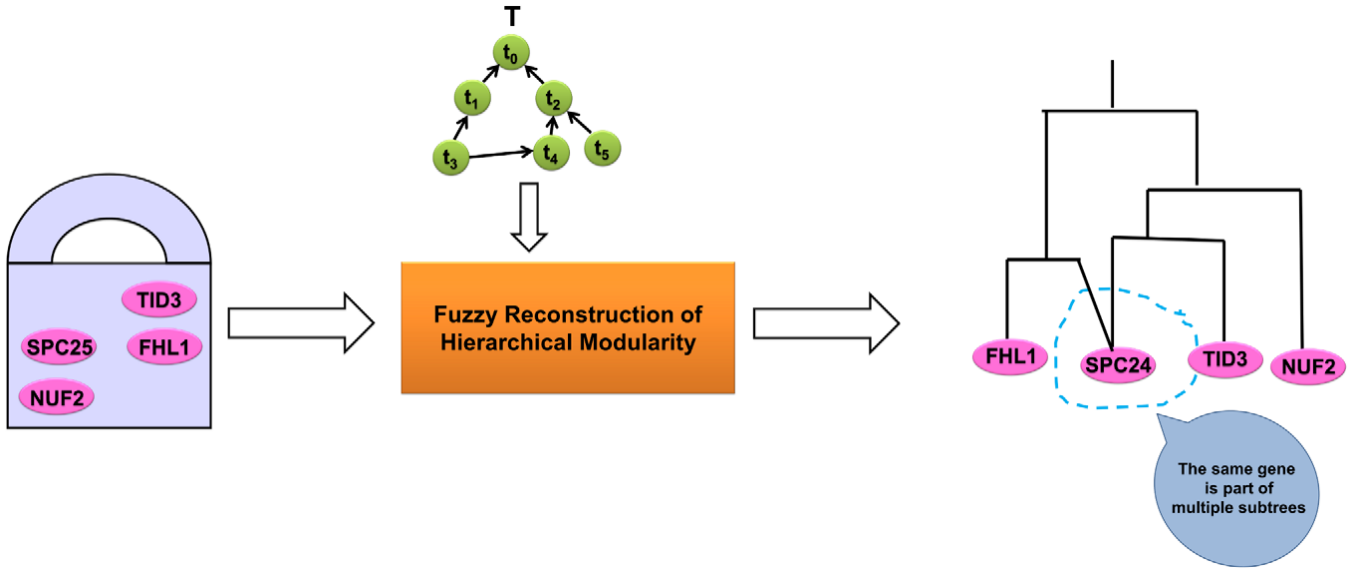
Overview of fuzzy reconstruction of hierarchical modularity.

Our method provides two main functionalities:

1. Given a hierarchical module and a hierarchical functional taxonomy, our method can assess the functional coherence of the module and provide a hierarchical functional annotation. The overview of this functionality is provided in Figure 4.
2. Given a module as a “bag of genes” and a hierarchical functional taxonomy, our method can build the functional hierarchy of the module, i.e, provide a global functional view of the module. The overview of this functionality is provided in Figure 5.

In the following subsections we discuss the technical details of the two functionalities.

### Hierarchical taxonomy of functional terms (HTFA)

Let 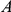 = {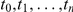}, 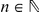, be a set of functional annotation terms. A functional annotation term (e.g., lyase activity) describes a function that a gene or a protein can carry out in the cell. A gene 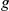 can be annotated with a subset 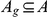 of functional annotation terms. If 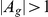, then 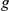 is multi-functional. If 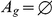, then 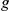 is called a hypothetical or unannotated gene. A functional term 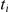 is more specific than a functional term 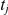, if it is a subtype of 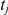. For example, lyase activity is a subtype of catalytic activity. Moreover, the same term can be a subtype of multiple terms. To capture functional specificity of terms, we will next define a hierarchical taxonomy of functional terms (HTFA).

**Figure 6:**
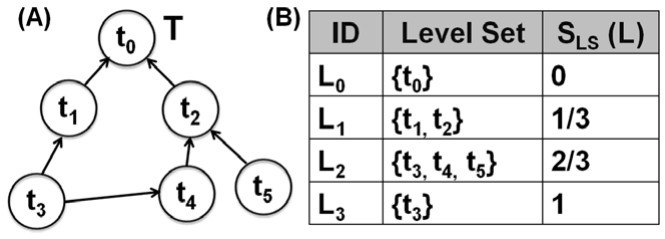
An illustration of a hierarchical taxonomy 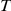 over the set of functional annotation terms 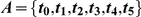. (A) A DAG view. (B) A level set view.

A hierarchical taxonomy 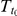 of functional terms 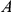 is a directed tree or a directed acyclic graph (DAG) with the set 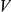 of labeled nodes (see Figure 6.A), such that

1. The labeling function 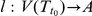 is a *bijection*, i.e., every node 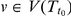 is labeled with only one term 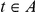, and each term 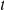 is assigned to only one node 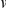, and
2. Label 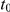 is assigned to only one node that is called the root node.

Whenever the context is clear, 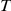 and 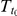 will be used interchangeably. Likewise, we will simply use 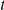 to refer to the node 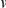 with label 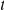 (i.e., 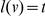).

Due to its hierarchical nature, 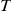 can be represented as a level set 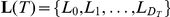 (see Figure 6.B), where 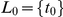 and level 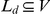 is a set of nodes visited at distance 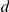 from the root 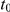 during the depth-first traversal of 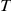, and 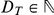 is the tree depth. Note that if 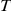 is a DAG (e.g., the Gene Ontology [10]), then 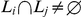 for some 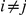. In other words, the node can occur at different levels in the taxonomy.

A pair of nodes 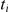 and 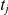 in 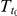 forms an *ancestor* relationship 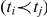, if there is a simple directed path from 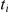 to 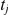 in 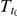. An ancestor relationship between a pair of nodes 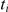 and 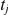 in 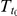 represents a *functional specificity* relationship, namely, a functional term of the child node is a subtype of the functional term of its parent, grandparent, grandgrandparent, and so on. This relationship is transitive, i.e, 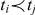 and 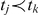 imply 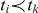. Also, a child can have multiple parents, as in a DAG.

Given this fact, we next introduce the *functional term specificity score* 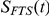 for a node 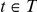 as follows:
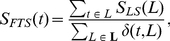
(1)where 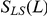 (see Figure 6.B) is the level specificity score associated with level 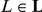 and defined as
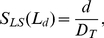
(2)and 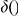 is a term characteristic function that specifies whether the term 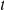 occurs at level 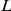:
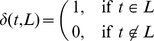
(3)


A distinct pair 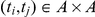 of functional terms is called *related*, if the corresponding nodes in 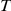 form an ancestor relationship, i.e., 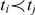. More generally, a set of terms 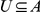 is called an *unrelated* set, or an *unrelated term set* in 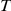, if no distinct pair 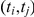, s.t. 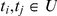, is related in 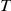.

Let 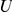 be an unrelated functional term set in 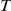. Then, as defined by Equation 4, the *ancestor functional term set* 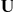 of 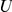 is the set of all the functional terms 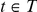 on any simple path from any node 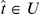 to the root node 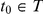:
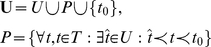
(4)For example, consider an unrelated functional term set 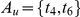 in Figure 6. According to Equation 4, its ancestor functional term set is 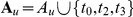.

### Hierarchical gene module (HGM)

Given a set of genes 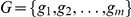, a hierarchical gene module (HGM) 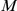 is an undirected tree over the set 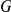 of leaf nodes. Given the hierarchical taxonomy 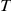 of functional terms 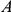, let an unrelated term set 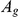, 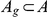, denote the functional annotation of gene 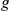.

#### Hierarchical functional annotation

Given an HTFA taxonomy 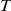 and an HGM module 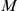 with a functional annotation 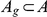 for each leaf node gene 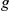, *hierarchical functional annotation* of 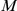 is the function 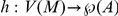 that maps each node 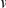 in 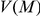 to the set 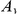 from the power set of 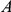 such that:

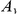
 is the set of the *most specific common functional terms* among 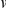
's children, and

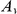
 is an unrelated functional term set in 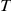.

Next, we will formally define the first condition, i.e., the set of the most specific common functional terms among the child nodes of 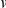. Let 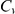 be the set of child nodes of 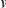. Note that if 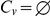, then 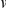 is a leaf node 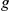 and 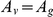. Otherwise, as defined by Equation 5, 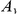 is derived from the intersection of the ancestor functional term sets of 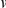
's children (see Equation 4) by maximizing the size of the unrelated functional term set 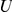 in the power set of this intersection:
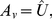
(5)

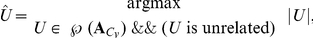


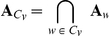



**Figure 7:**
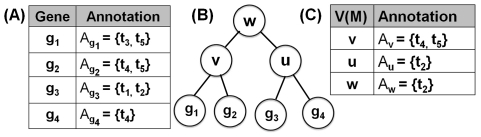
Hierarchical functional annotation of a gene module 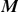 for a gene set 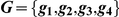 **given the taxonomy** 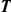 in Figure 6. (A) Functional annotation of genes in 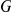 by unrelated term sets in 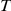. (B) A hierarchical gene module 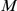. (C) The resulting annotation of the internal (non-leaf) nodes in 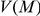.

For a hierarchical functional module 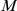 in Figure 7 and a taxonomy 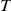 in Figure 6, consider 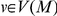 as an example with 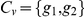 and 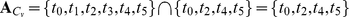. Then the maximum size unrelated term set in the power set of this intersection defines the functional annotation set 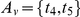 for the internal node 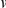.

#### Functional coherence

Existing functional coherence analysis techniques analyze the input functional module in its entirety without considering its hierarchical structure. Additionally, most methods depend on a *reference set* by incorporating its annotation distribution and size into their scoring formula [20]–[22]. A reference set is a group of proteins that forms a superset of the functional module. Khatri *et al.* discuss the difficulties with selecting the right reference set [64].

Here, we introduce a method that accounts for the hierarchical structure of the module 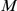 when determining its functional coherence. Additionally, the scoring function does not directly rely on any reference set. More specifically, given the hierarchical gene module 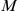 and its functional annotation, the functional coherence score, called *hierarchical modularity score* (HMS), 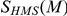 of 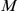 is defined by Equation 6:
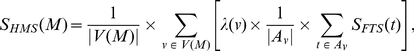
(6)where the functional term specificity 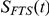 is defined by Equation 1, and the *penalization factor* 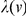 is discussed in the following section.

#### Penalization factor (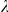)

**Figure 8:**
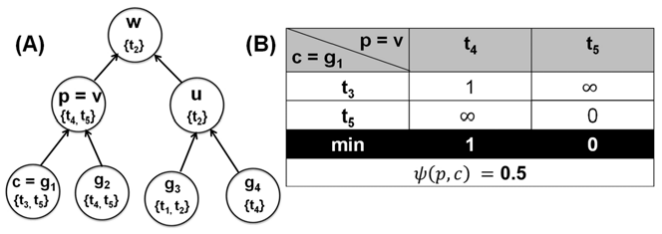
Illustration of penalization factor calculation. (A) Hierarchical annotation of the functional module defined in Figure 7. (B) Dissimilarity score 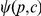 for a parent 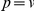 and a child 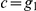.

Consider a hierarchical gene module 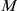 with its hierarchical functional annotation (see Figure 8.A), as described in *Hierarchical functional annotation* section. Let 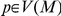 be a parent node with its children 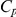. Given the functional annotation term sets, 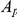 and 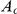, for the parent 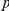 and its child 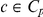, respectively, a dissimilarity score 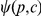 between 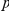 and 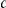 is defined by Equation 7 (see Figure 8.B):
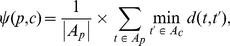
(7)where the distance 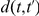 is the length of the shortest simple path (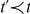) from node 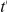 to 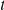 in 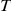, or 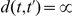, if 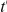 and 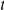 are not related.

**Figure 9:**
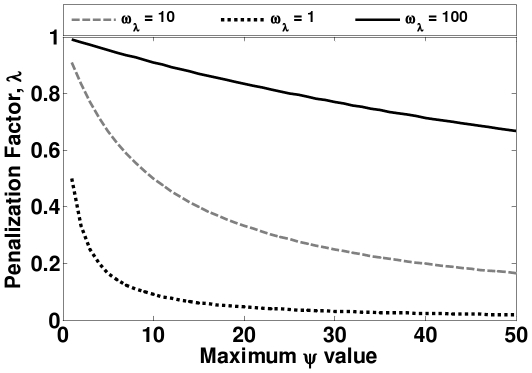
Comparison between the three penalization factor functions considered.

Given Equation 7, the penalization factor 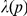 for the parent node 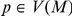 is then defined by Equation 8:
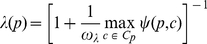
(8)For the example in Figure 8, 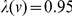 for 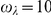, because the dissimilarity scores between 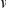 and its children 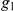 and 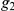 are 0.5 and 0, respectively. Also, Figure 9 depicts the behavior of 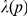 for different values of 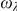 in Equation 8, as the maximum value of 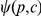 varies from zero to its maximum possible value of the tree depth 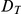 in the taxonomy 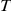. If 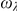 increases from one to 100, then node score's penalty decreases from 50% (even for immediate neighbors in 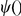) to 13% (for the largest taxonomy depth 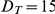 in the Gene Ontology [10]). More information on choosing 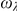 values can be found i *Choosing* 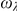 *value* section.

### Assessing statistical significance

To provide a robust assessment of statistical significance for 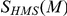, we measure an empirical 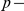
value for 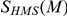 score assigned to each hierarchical module 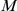 using the Monte Carlo procedure described in [32]. Specifically, for hierarchical module 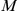 over a set of 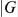 genes from organism 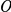, we randomly sample 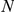 subsets of size 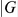 from the entire genome of organism 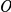, build the hierarchy, and compute the 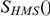. Then, we estimate an empirical 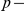
value for 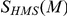 as 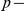
value = 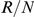, where 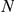 is the total number of random samples (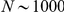) and 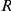 is the number of the samples that produce a test statistics 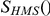 greater than or equal to the 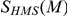.

### Fuzzy reconstruction of hierarchical modularity

In *Hierarchical gene module* section, the hierarchical structure for a gene module 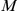 was provided as an input. Based on this structure and the hierarchical taxonomy of functional annotation terms (*Hierarchical taxonomy of functional terms* section), we provided means both for inferring 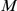
's hierarchical functional annotation (*Hierarchical functional annotation* section) and for estimating 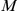
's functional coherency via hierarchical modularity scoring (*Functional coherence* section).

In contrast, here we consider a somewhat inverse problem, namely, the reconstruction of a hierarchical structure of a functional module 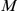 defined by its gene set 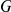. 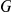 is often referred as a “bag of genes.” On the one hand, it seems that any hierarchical clustering method could be used to reconstruct the hierarchical functional modularity from such a “bag of genes.” On the other hand, the presence of multi-functional genes suggests that the same gene could belong to multiple subtrees in the hierarchy–the property that is not often guaranteed by any hierarchical clustering method. Therefore, we will first need to introduce “fuzziness” into the process of building a functional hierarchy for 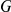. For example, in Figure 5, a bag of genes containing *SPC24*, *TID3*, *NUF2*, and *FHL1* and a functional annotation taxonomy 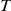 are provided as input to the method. It is known that *SPC24*, *TID3*, and *NUF2* are functionally related because they are part of the Ndc80 protein complex but *SPC24* is also trnascriptionally regulated by *FHL1* [83] and so *SPC24* is part of multiple subtress and, hence, fuzziness is introduced.

Existing fuzzy clustering schemes typically introduce some fuzziness into some known clustering algorithm. C-means [84], [85] is a typical example of this kind. Others are typically partitional by nature [27], [28], [86]. Agglomerative fuzzy clustering algorithms are not common, because agglomerative techniques are considered “hard clustering,” i.e., it becomes difficult to move an element from an existing cluster to a new cluster. Ideally, any fuzziness in a clustering procedure should be introduced, while the hierarchy is being built and not as a post-processing step.

To meet these requirements, we propose a *taxonomy-based, agglomerative, fuzzy inference* (TAFI) of the hierarchical gene module 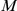 from a gene set 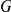, provided each gene 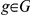 is annotated with an unrelated functional term set 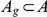 in a hierarchical taxonomy 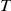 of functional annotation terms 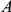 (see *Hierarchical taxonomy of functional terms* section). The overview of this method is provided in Figure 5.

Similar to an agglomerative hierarchical clustering (AHC) process, TAFI starts with assigning each gene to its own cluster and proceeds building the hierarchy in an iterative, bottom-up manner, but it introduces fuzziness by allowing multiple cluster pairs to merge simultaneously at each iteration. The two user-defined parameters control this fuzziness process at each iteration: (a) the *merging factor* 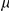 and (b) the *stopping criterion* 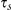. The former defines what cluster pairs get merged at a given iteration 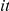. Namely, unlike traditional AHC that merge the pair of clusters with the maximum similarity 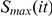, TAFI allows for clusters with suboptimal similarity to be merged as well. Suboptimality is defined by the percentage 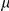 of 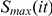. In addition, TAFI prevents the formation of unrelated clustering modules by stopping the bottom-up cluster merging process at iteration 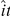 as soon as 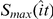 value falls below 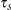.

Note that Horng *et al.* [87] proposed to merge the cluster pairs whose similarity is greater than 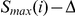 unlike TAFI ‘s way of restricting to 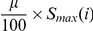 similarity threshold. The reason behind our choice of multiplicative factor rather than additive/subtractive factor is the following. If 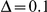 and 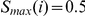, then any cluster pair with inter-cluster similarity greater than 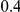 would be merged. The value of 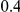 is 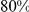 of 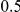. However, if 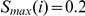, then any cluster pair with inter-cluster similarity greater than 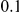 would be merged, but the value of 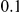 is only 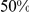 of 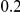. The criterion becomes more stringent with a larger value of 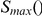 and, conversely, it becomes more lenient, as 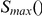 gets smaller. In contrast, our choice of the merging factor allows for resolving this inconsistency issue.

Also, observe that multiple merges at each iteration can sometimes result in the same subtree being formed repeatedly. This leads to redundancy. Thus, TAFI employs pruning, where a merge is allowed only if the merge results in a subtree that has not been already formed.

In addition, we need to make two important decisions in order to apply TAFI: (1) the inter-cluster similarity measure and (2) the linkage algorithm. For the inter-cluster similarity measure, we use Equation 6 that calculates the hierarchical modularity score 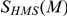 for a hypothetical module 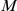 that could be formed if the two clusters, or hierarchical tree modules 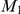 and 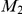, were merged by adding a new root node 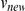 and making the root nodes 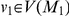 and 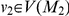 to be the children of 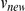.

It is worth noticing that 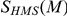 is a semi-metric, and this property has direct implications on our choice of the base clustering algorithm. Since semi-metrics do not adhere to the triangle inequality principle, we can resort to an average, single, complete, or centroid linkage algorithm as our base clustering technique. Therefore, the effective clustering techniques, such as Ward's method cannot be used in conjunction with semi-metrics [88].

## Supporting Information

Supplement S1 Results discussed in *Functional coherency of protein functional modules* and *Functional coherency of protein pairs* sections.
(XLSX)

Supplement S2 Results discussed in *Consistency analysis for KEGG metabolic pathways* and *Consistency analysis for MIPS protein complexes* sections.
(RAR)
